# Selection of the Optimal Method for Creating Various Forms of Biocompatible Xenodermal Materials

**DOI:** 10.17691/stm2022.14.1.04

**Published:** 2022-01-28

**Authors:** К.I. Melkonyan, Y.А. Kozmai, А.А. Verevkin, Т.V. Rusinova, А.S. Asyakina, М.L. Zolotavina

**Affiliations:** Associate Professor, Head of the Central Research Laboratory; Kuban State Medical University, 4 Mitrofana Sedina St., Krasnodar, 350063, Russia; Junior Researcher, Central Research Laboratory; Kuban State Medical University, 4 Mitrofana Sedina St., Krasnodar, 350063, Russia;; Researcher, Central Research Laboratory; Kuban State Medical University, 4 Mitrofana Sedina St., Krasnodar, 350063, Russia;; Researcher, Central Research Laboratory; Kuban State Medical University, 4 Mitrofana Sedina St., Krasnodar, 350063, Russia;; Assistant Researcher; Kuban State Medical University, 4 Mitrofana Sedina St., Krasnodar, 350063, Russia; Graduate Student, Department of Genetics, Microbiology, and Biochemestry; Kuban State University, 149 Stavropolskaya St., Krasnodar, 350040, Russia; Associate Professor, Department of Genetics, Microbiology, and Biochemestry; Kuban State University, 149 Stavropolskaya St., Krasnodar, 350040, Russia

**Keywords:** xenodermal biomaterials, surgical biomaterials, dermis, alkaline hydrolysis, decellularization

## Abstract

**The aim of the study** was to select the optimal method for creating surgical porcine dermis-based biomaterials and to assess their biological safety.

**Materials and Methods:**

To create xenodermal biomaterials, the native skin of a 4-month-old Landrace pig was used. The porcine dermis was processed with saline (protocol No.1), peroxide-alkaline (protocol No.2), and alkaline (protocol No.3) solutions. The obtained samples were stained with hematoxylin-eosin and a DAPI fluorescent dye. Quantitative DNA analysis and assessment of cytotoxicity by the LIVE/DEAD assay were also performed. Samples were implanted/injected subcutaneously to 6-month-old male Wistar rats (n=30) weighing 260±20 g and explanted on day 14 of the experiment. Histological sections were stained with hematoxylin-eosin. Computer morphometry was performed using GraphPad Prism v. 6.04.

**Results:**

Samples of surgical materials obtained according to the three protocols had different physical characteristics: dermis treated according to protocol No.1 was dense and white in color after processing; samples processed by protocol No.2 were transparent and dense, and samples treated according to protocol No.3 had transparent gel-like structures. Histological analysis has shown oxyphilicity and extracellular matrix structure loss in all samples, and DAPI staining has revealed the destruction of cell nuclei. Nevertheless, DNA amount in the samples processed according to protocol No.1 did not meet the established quality criterion for decellularization (50 ng/mg dry weight). Further cytotoxicity assessment *in vitro* and *in vivo* was carried out only for samples fabricated according to protocols No.2 and No.3. According to the LIVE/DEAD analysis, both samples were not cytotoxic. On day 14 after the subcutaneous sample implantation, no signs of suppuration and immune rejection were found in the animals.

**Conclusion:**

To obtain surgical materials in the form of bioplastic coatings, it is recommended to use alkaline-peroxide treatment of the dermis, while hydrogel coatings are produced by alkaline hydrolysis.

## Introduction

Presently, the development of various reconstructive materials used as wound coatings and surgical implants in the diseases of different etiology remains urgent [1]. The existing standards in surgery imply application of patient native tissues to replace the defects resulting undoubtedly in better effect than xenogenic materials [2]. However, an additional volume of surgical intervention, traumaticity, and difficulty in fixing the self-tissues restrict the application of this method inspiring search for novel biocompatible and biodegradable materials for solving a wide spectrum of clinical tasks.

The modern pharmaceutical market offers different forms of biomaterials depending on the specificity of their application. For example, there are products presented in the form of matrices, lyophilisates, powders, films, hydrogels, sponge materials, and patches [3, 4]. The functional feature of many surgical materials is a complex favorable effect on tissues: they may be carriers of medicinal agents and deliver biologically active substances, growth factors, and regeneration stimulators to the damaged zone [4–6].

Surgical materials which are analogs to the extracellular matrix (ECM) and imitating cellular microenvironment are being actively investigated and implemented into clinical practice: synthetic scaffolds fabricated from polymer substrates (for example, polycaprolactone, polyethylene glycol, and polyglycolic acid); hydrogels synthesized from cross-linked hydrophilic polymers (for example, polyacrylic acid, polyethylene glycol, and polyvinyl alcohol); ceramic-based scaffolds made from hydroxyapatite or tricalcium phosphate. There are also materials based on the natural biopolymers from animal and plant raw materials: alginates, chitosan, chondroitin sulphates, cellulose, gelatin, dextrin, silk fibroin, and collagen [7, 8]. The disadvantage of synthetic materials is their inability to biodegradation and frequent postoperative complications (infiltrates, abscesses, fistulas, pyoinflammatory processes). Biological materials based on the connective tissue ECM produce minimal local inflammatory reaction and provide natural microenvironment for functional tissue regeneration [9]. It is worth mentioning that biocompatibility and structural similarity of the biological materials with the native ECM makes them eligible for using as supporting and replacing implants as well as for acceleration of tissue regeneration.

Biological materials can be produced by soft lithography, electrospinning, 3D printing, or tissue decellularization [8]. The most preferred method is a decellularization technology which makes it possible to preserve the histostructure of the collagen-containing xeno- and allogenic tissues [10]. Chemical methods of decellularization are the most optimal as they promote maximal cell removal with minimal damage to ECM, preservation of its three-dimensional ultrastructure, spatial topology, and chemical composition. It should be also underlined that decellularization by means of chemical agents is usually considered a cost-effective technique, but a relatively long period of treatment may be a substantial problem for optimization of production time for these materials [11]. Decellularization procedures based on collagen-containing tissue processing with alkaline solutions are also popular enough [12]. Decellularized matrices for medical applications are most frequently fabricated from xenogenic tissues [13]. Therefore, the procedure of manufacturing purified ECM must provide absence of xenotransplant rejection after its implantation into the patient tissues.

Thus, with all the above said it may be concluded that it is necessary to develop biological surgical materials with tissue-specific compatible matrix capable of biodegradation and optimizing wound regeneration.

**The aim of the study** was to select the optimal method of creating surgical biomaterials and to assess their biological safety.

## Materials and Methods

### Sampling and decellularization of dermis

Xenodermal materials were derived from the native skin of a 4-month-old Landrace pig. The animal was injected with lethal doses of Zoletil and Xylazine. The epidermis was removed from the donor area of the skin using an electrodermatome with a 100-mm disc blade diameter, thereafter, 0.50±0.05 mm-thick samples of dermis weighing 0.50±0.03 g were obtained. Samples underwent chemical decellularization using three known techniques with the following modifications: protocol No.1 — treatment with concentrated saline solutions [14], protocol No.2 — treatment with alkaline and hydrogen peroxide solutions [15], protocol No.3 — treatment with an alkaline solution [15].

According to protocol No.1, the samples were in the supersaturated solution containing 1.19 M KCl, 1.74 M NaCl, and 0.86 M CaCl2 (Reachem, Russia) for 96 h at 25°С, hydromodule (tissue:solution) 1:3. Excessive salts were removed by successive washing in a 0.3% boric acid solution (Reachem), deionized water, EDTA solution (Thermo Fisher Scientific, USA), and finally, the samples were washed in deionized water until pH stabilization.

In compliance with protocol No.2, the dermis samples were processed with a mixture of 5% NaOH solution (Vekton, Russia) and 3% H2O2 solution (Iodine Technologies and Marketing, Russia) in 1:1 ratio at 25°С for 48 h (hydromodule 1:5). Then the samples were washed in deionized water until pH was stabilized.

Following protocol No.3, the samples were treated with 5% NaOH for 12 h (hydromodule 1:5). Stabilization of pH was reached by washing the samples with deionized water.

All dermis samples underwent a routine histological analysis before and after the treatment to assess the ECM structure preservation.

### Quantitative DNA analysis.

After treatment of the dermis, DNA quantity was determined by DNeasy Blood & Tissue Kit (QIAGEN, Sweden) following the manufacturer’s protocol using NanoDrop ND-1000 spectrophotometer (Thermo Fisher Scientific).

### Assessment of cell nuclear destruction using DAPI staining.

DAPI staining was performed in the following way: 4–5-μ paraffin sections of the obtained materials were fixed with 4% formaldehyde for 10 min. Then DAPI (Sigma-Aldrich, USA) in 1:1000 dilution was added to the samples and incubated for 5 min.

### Assessment of the biomaterial cytotoxicity by the LIVE/DEAD method

To perform the LIVE/DEAD assay (LIVE/DEAD Cell Imaging Kit, Thermo Fisher Scientific), a line of human dermal fibroblasts DF-1 received from the Russian collection of cell cultures (Institute of Cytology, Russian Academy of Sciences, Saint Petersburg, Russia) was used. The cells were incubated in the DMEM medium (Gibco, England) with the biomaterial samples for 24 h, then stained with fluorescent dyes such as calcein-AM and ethidium homodimer: the live cells exhibited green fluorescence while the dead cells produced red fluorescence. Olympus cellSens Entry software (Olympus, Japan) provided visualization of the fluorescence.

### Subcutaneous tests

Subcutaneous implantation/injection of the biomaterial samples was made to the 6-month-old male Wistar rats (n=30) weighing 260±20 g. Animals were anaesthetized with 14 ml/kg zoletil 100 solution and 1.2 ml/kg sedamidin solution and then the tested materials were injected under the skin in the interscapular area. Antimedine solution (20 mg/kg) was used to get out the rats from anaesthesia. On day 14 after the subcutaneous biomaterial tests, the samples were explanted and morphologically and histologically analyzed.

### Microscopy.

Microscopic, histological, and fluorescent investigations were conducted in three visual fields/sections for each sample using Olympus СХ41 microscope (Olympus), data and images were processed using Olympus cellSens Entry software.

The study was approved by the Independent Ethical Committee of Kuban State Medical University. All manipulations were done in compliance with the requirements of Order No.708n of the Russian Ministry of Health of August 23, 2010 “On the approval of the rules of laboratory practice” and the Federal Law “On the protection of animal cruelty” of December 1, 1999.

***Statistical processing of data*** on morphometry of biomaterial samples and quantitative DNA analysis was performed using GraphPad Prism v. 6.04. To check the character of value distribution, Shapiro–Wilk test was applied. Since the distribution did not differ from normal, the results were presented as М±S, where М is arithmetic mean, S is standard deviation. Student’s t-test was used to compare the DNA content in the samples of different biomaterials. Differences were considered significant at p≤0.05.

## Results

**Figure 1:**
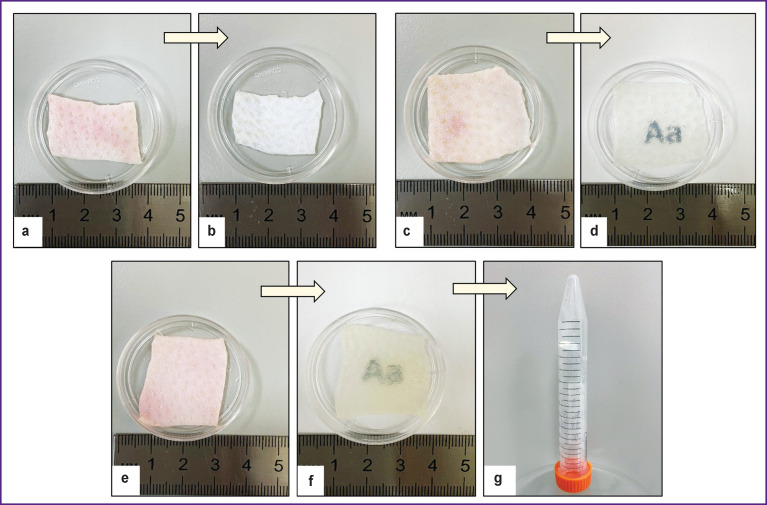
Appearance of samples before (а) and after (b) treatment according to protocol No.1; before (c) and after (d) treatment according to protocol No.2; before (e) and after (f), (g) treatment according to protocol No.3

The samples from biological material fabricated according to protocol No.1 with a concentrated saline solution were white in color, strong enough, did not crush when pressed by forceps (Figure 1 (a), (b)). The average thickness of the samples was 1.0±0.05 mm. The samples treated according to protocols No.2 and No.3 became semitransparent after pH stabilization (Figure 1 (c)–(f)). The average thickness of the samples processed according to protocol No.2 was 1.0±0.05 mm as well. The dermis samples treated following protocol No.3 formed a gel-like structure 12 h later (Figure 1 (g)).

**Figure 2:**
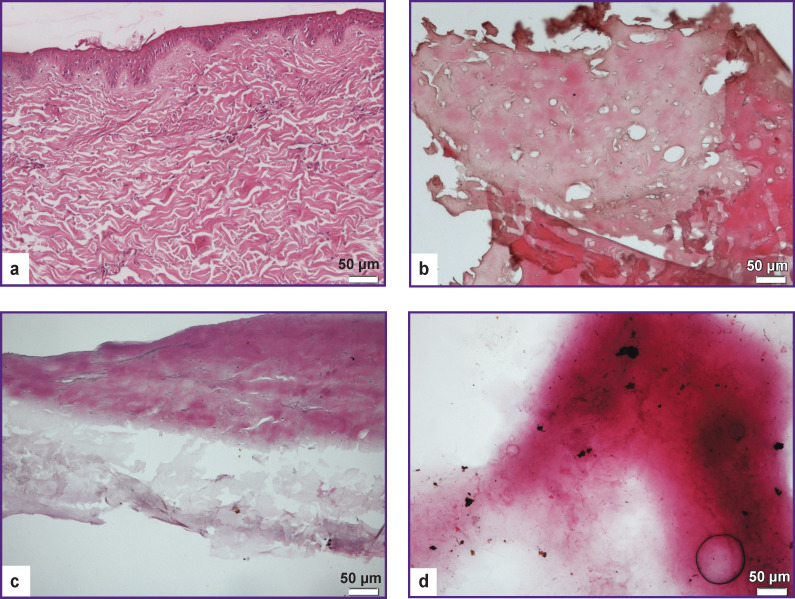
Histological analysis of native dermis samples (а), after treatment according to protocol No.1 (b), protocol No.2 (c), and protocol No.3 (d); ×100

The histological analysis has shown that the dermis samples obtained in compliance with protocols No.1 and No.2, in contrast to the native tissue (Figure 2 (a)), looked like an oxyphilic mass presented by multidirectional cords of collagen fiber bundles, the structure of which was predominantly homogeneous (Figure 2 (b), (c)). The material processed according to protocol No.3 also represented an oxyphilic structure, in which the most marked hydrolysis and swelling of the polymers were observed (Figure 2 (d)).

**Figure 3:**
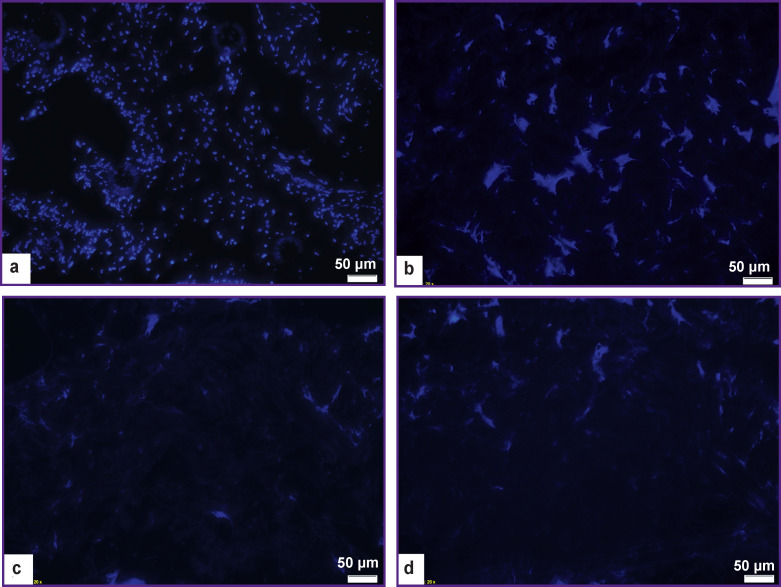
Results of DAPI staining of native dermis samples (а), after treatment according to protocol No.1 (b), protocol No.2 (c), and protocol No.3 (d); ×200

The DAPI staining has shown absence of cell nuclei in all samples proving the effectivity of the decellularization procedures and perhaps better compatibility of the materials when they are used (Figure 3).

**Figure 4:**
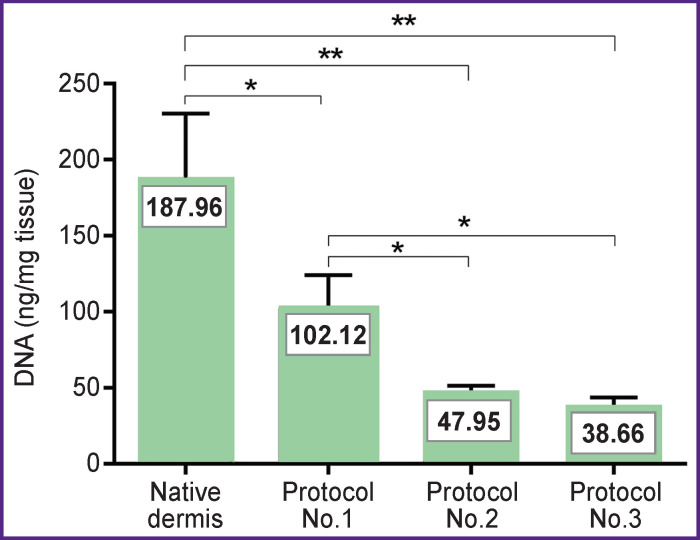
Results of quantitative DNA analysis: * p<0.05; ** p<0.01

A quantitative DNA analysis has been performed to determine optimal conditions and effectivity of the decellularization process (Figure 4). The investigation has demonstrated that the amount of DNA in the samples produced according to protocols No.2 and No.3 decreased statistically significantly up to 25.51% (47.95±2.03 ng/mg dry substance; p≤0.05) and 20.57% (38.66±1.64 ng/mg dry substance; p≤0.05), respectively, relative to the DNA content in the native dermis (187.96±5.21 ng/mg dry substance — 100%). While the DNA content in the samples processed according to protocol No.1 amounted to more than 50 ng per 1 mg dry weight which did not satisfy the known criterion of decellularization quality [16] and therefore these samples did not undergo further investigations.

**Figure 5:**
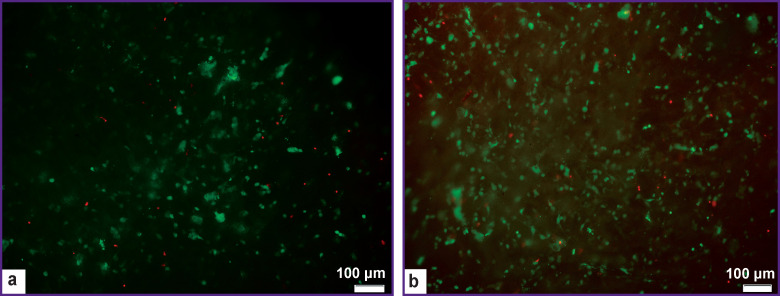
Assessment of biomaterial cytotoxicity: (a) protocol No.2; (b) protocol No.3; *green staining* — live cells, *red staining* — dead cells; ×100

The analysis of data obtained by the LIVE/DEAD method has demonstrated a large number of viable cells after the co-cultivation of the samples fabricated according to protocols No.2 and No.3 and dermal fibroblasts indicating that there was no cytotoxic effect of the tested matrix samples (Figure 5).

**Figure 6:**
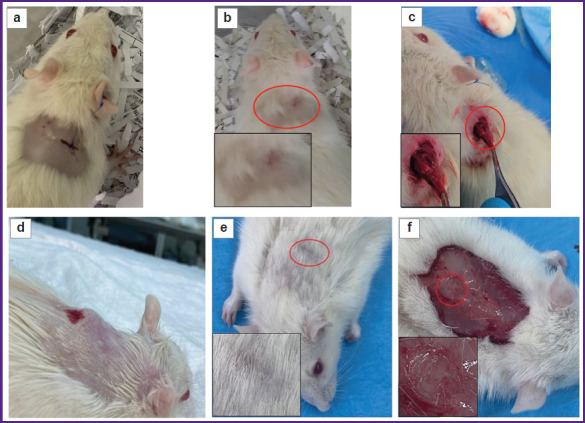
Results of subcutaneous tests, day 14: (а)–(c) protocol No.2; (d)–(f) protocol No.3

The results of the subcutaneous tests of the experimental materials are presented in Figure 6. All the animals did not have macroscopic signs of inflammation on day 14 at the site of sample introduction after the dermis treatment according to protocols No.2 and No.3, no suppuration and soft-tissue edema in the implantation area were observed (Figure 6 (b), (e), (f)). In animals with the subcutaneous implantation of the biomaterial samples prepared according to protocol No.2, a connective tissue capsule was formed though no fixation of the implant to the surrounding tissues was noted (Figure 6 (c)).

**Figure 7:**
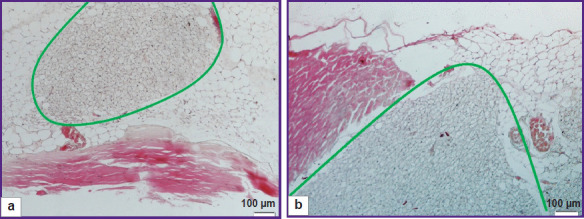
Staining with hematoxylin and eosin, day 14: (а) protocol No.2; (b) protocol No.3. Green lines show the borders of the samples and surrounding tissues; ×100

The histological assessment of the tissue reaction to the implantation of the samples obtained in compliance with protocols No.2 and No.3 has established that no signs of inflammation and formation of connective tissue capsules at the site of implantation of the tested dermal samples were not observed on day 14 (Figure 7).

The dermal materials were characterized by partial biodegradation, were insignificantly impregnated with fibrous exudate which signifies the possibility of their application as a basis for regeneration and biointegration into the tissues in surgical treatment.

## Discussion

At present, there are data on the modern technologies for tissue decellularization using alkaline hydrolysis for cartilage tissue, amniotic membrane, and intestinal submucosa; however, investigations on the porcine dermis treatment by the alkaline technology are rather scanty and contradictory [17, 18]. The authors of this article have developed mono- and polycomponent methods of alkaline and saline processing of the primary products for fabricating various forms of dermal ECM and also performed a complex assessment of the biological compatibility and safety of the obtained materials. There are single issues devoted to the complex assessment of biocompatibility and safety of the acellular dermal materials for medical practice. For example, Li et al. [19] conducted similar investigations but in relation to the fish skin-based biological materials. Hoganson et al. [20] proposed the method of obtaining acellular dermal matrix on the basis of the porcine dermis possessing significant biological activity and safety in relation to cellular cultures which correlates with the data obtained by us.

Treatment with detergent solutions has been known as one of the most effective methods of producing decellularized ECM from dense fibrous connective tissues [18]. However, most detergents (Triton X-100, sodium deoxycholate, sodium dodecyl sulfate, CHAPS) [16] are rather expensive, additional treatment with enzymes (nucleases, trypsin, dispases, and others) which increases production cost of the fabricated material is required. The technology of derma processing developed by us is based on the relatively inexpensive chemical detergents such as hydrogen peroxide, solutions of mineral salts and alkalis. The proposed algorithms are simple to use as do not imply multi-step treatment and allow the production of biologically safe materials.

The quantitative analysis of the DNA content has shown that despite a high extent of destruction of the native dermis histoarchitectonics and similar histomorphological picture for all samples of the created biological materials, processing with concentrated saline solutions does not fully remove the nuclear material of the dermis cells (protocol No.1). A high DNA content in the samples treated with the concentrated saline solutions may be explained by the fact that only impairment of cellular membranes with partial destruction of the molecules integrated into the cells occurs during this process causing the necessity of additional enzymatic treatment [16]. To increase the extent of dermis decellularization, it is necessary to modify protocol No.1 either by adding detergents (Triton X-100, sodium dodecyl sulfate, sodium deoxycholate, etc.) or by increasing the time and number of cycles for material handling. This conclusion is in line with the investigations [20, 21], in which a multi-step processing with organic solvents, detergents, saline solutions, and enzymes was employed but DNA values in this case did not exceed 50 ng/mg tissue.

Alkaline solutions cause effective hydrolysis and solubilization of the cellular components as well as proteins and ECM glycoproteins, the presence of which in the matrix may lead to the undesirable response of the recipient’s organism [22].

After alkaline peroxide treatment, samples represented different structural materials: bioplastic material and hydrogel. The difference in the form of the biomaterials is likely to be connected with swelling of the collagen fiber bundles during treatment with alkaline solutions. The process of swelling is caused by ionization of the side groups of tropocollagen chains in the solution with a high pH value as well as dissociation of the glycosaminoglycan molecules bound to the collagen. Swelling results in the increase of the distance between the collagen fiber bundles, and the collagen network loosens. However, a high extent of hydrogen peroxide oxidizing capability also causes a definite action. Rather a high concentration (3%) of hydrogen peroxide enhances the strength characteristics of the obtained bioplastic material not permitting a high degree of collagen hydrolysis. This correlates with the literature data: at relatively high concentrations of hydrogen peroxide (>0.5%) collagen materials demonstrate the increase of ultimate tensile strength [11, 23]. There also may be processes of destruction and wash-out of glycosaminoglycans composing glycoproteins participating in the packaging of collagen fibers into bundles. Thickening and structural homogeneity of the collagen bundles due to its swelling are likely to increase its density. Nevertheless, matrices after peroxide-alkaline treatment are supposed to promote better cell viability for successful connective tissue regeneration due to enlargement of the surface area connected with loosening of the ECM fibrous network and may be used as supporting implants and wound dressings. In the known study by Schwarz et al. [24], human and porcine cartilage tissue underwent decellularization using alkali (1N NaOH solution) and hydrogen peroxide (5% H2O2 solution); guanidine hydrochloride and sodium acetate were applied additionally. The samples looked like a dense bioplastic material and had a characteristic white color that disagrees with the data obtained by us.

Alkaline treatment without hydrogen peroxide resulted in formation of hydrogel collagen-containing material which may be explained by collagen structure impairment, i.e. cleavage of the collagen fibrils and destruction of unrestorable collagen cross-links. In the analogous study [11], the dermis was treated with 0.06 М NaOH, however, the time of treatment became much longer due to a low concentration of the alkali, besides the produced material was almost similar to the bioplastic material by its structure. In the other investigation [25], bovine dermis was processed with 1 М NaOH solution for 20 h. The histological analysis did not register cellular cultures, while the quantitative DNA analysis showed 13.1 ng/mg tissue which correlates with our results, however, the authors failed to obtain a gel-like structure of the material. The development of connective tissue-based hydrogels has been described in several researches, but it implies a long-term and labor-consuming technology including decellularization, shock freezing, lyophilic drying, crushing in the cryomill, and swelling [26, 27]. Our method is simple, single-component, and the fabricated material possesses satisfactory biocompatibility and biosafety.

## Conclusion

The proposed decellularization protocols allow fabrication of various forms of biologically safe nontoxic biomaterials which may be used for guided tissue regeneration in surgical practice. To create bioplastic coatings, it is recommended to use alkaline-peroxide treatment which provides formation of a dense semitransparent material convenient for use and observation of the healing process. Application of alkaline hydrolysis is considered optimal for creation of hydrogel coatings and injection materials. However, long-term investigations are needed to study the interaction of biomaterials with the tissue in more detail.
